# The normality assumption on between-study random effects was questionable in a considerable number of Cochrane meta-analyses

**DOI:** 10.1186/s12916-023-02823-9

**Published:** 2023-03-29

**Authors:** Ziyu Liu, Fahad M. Al Amer, Mengli Xiao, Chang Xu, Luis Furuya-Kanamori, Hwanhee Hong, Lianne Siegel, Lifeng Lin

**Affiliations:** 1 Department of Statistics, Florida State University, Tallahassee, FL USA; 2 Department of Mathematics, College of Science and Arts, Najran University, Najran, Saudi Arabia; 3 Department of Biostatistics and Informatics, University of Colorado Anschutz Medical Campus, Aurora, CO USA; 4 Ministry of Education Key Laboratory for Population Health Across-Life Cycle & Anhui Provincial Key Laboratory of Population Health and Aristogenics, Anhui Medical University, Anhui, China; 5 School of Public Health, Anhui Medical University, Anhui, China; 6 UQ Centre for Clinical Research, Faculty of Medicine, University of Queensland, Herston, Australia; 7 Department of Biostatistics and Bioinformatics, School of Medicine, Duke University, Durham, NC USA; 8 Division of Biostatistics, University of Minnesota School of Public Health, Minneapolis, MN USA; 9 Department of Epidemiology and Biostatistics, Mel and Enid Zuckerman College of Public Health, University of Arizona, Tucson, AZ USA

**Keywords:** Cochrane Library, Effect measure, Heterogeneity, Meta-analysis, Normality assumption, Q–Q plot

## Abstract

**Background:**

Studies included in a meta-analysis are often heterogeneous. The traditional random-effects models assume their true effects to follow a normal distribution, while it is unclear if this critical assumption is practical. Violations of this between-study normality assumption could lead to problematic meta-analytical conclusions. We aimed to empirically examine if this assumption is valid in published meta-analyses.

**Methods:**

In this cross-sectional study, we collected meta-analyses available in the Cochrane Library with at least 10 studies and with between-study variance estimates > 0. For each extracted meta-analysis, we performed the Shapiro–Wilk (SW) test to quantitatively assess the between-study normality assumption. For binary outcomes, we assessed between-study normality for odds ratios (ORs), relative risks (RRs), and risk differences (RDs). Subgroup analyses based on sample sizes and event rates were used to rule out the potential confounders. In addition, we obtained the quantile–quantile (Q–Q) plot of study-specific standardized residuals for visually assessing between-study normality.

**Results:**

Based on 4234 eligible meta-analyses with binary outcomes and 3433 with non-binary outcomes, the proportion of meta-analyses that had statistically significant non-normality varied from 15.1 to 26.2%. RDs and non-binary outcomes led to more frequent non-normality issues than ORs and RRs. For binary outcomes, the between-study non-normality was more frequently found in meta-analyses with larger sample sizes and event rates away from 0 and 100%. The agreements of assessing the normality between two independent researchers based on Q–Q plots were fair or moderate.

**Conclusions:**

The between-study normality assumption is commonly violated in Cochrane meta-analyses. This assumption should be routinely assessed when performing a meta-analysis. When it may not hold, alternative meta-analysis methods that do not make this assumption should be considered.

**Supplementary Information:**

The online version contains supplementary material available at 10.1186/s12916-023-02823-9.

## Background

The normality assumption is commonly used in most meta-analytic methods [1–4], but this assumption could be questionable in practice [5]. Specifically, the normality assumption is typically involved in two levels of meta-analysis with random-effects models. At the within-study level, two-stage meta-analysis methods assume the observed summary statistics follow normal distributions with an underlying true mean [6, 7]. This is generally valid if the sample sizes of individual studies are sufficiently large by the central limit theorem [5]. One-stage meta-analysis methods could avoid the normality assumption at this level by using exact distributions for outcome measures, such as the binomial likelihood for binary outcomes [8–10]. The validity of the within-study normality assumption could be affected by multiple factors, such as individual studies’ sample size, event probabilities of binary outcomes, and true distributions of continuous measures [11–14]. As such, this assumption needs to be evaluated on a case-by-case basis and is generally difficult to assess. If strong evidence indicates this assumption is violated, researchers should consider alternative meta-analysis methods (e.g., one-stage models) that do not make this assumption [8, 10, 15].

On the other hand, the normality assumption at the between-study level is typically required by the most commonly used one- and two-stage random-effects methods and is not guaranteed for large sample sizes by the central limit theorem. It assumes that the true effects of individual studies differ due to heterogeneity, and they follow a normal distribution with a mean of overall effect and variance of between-study heterogeneity [16]. This article will focus on this *between-study normality assumption*. Heterogeneity between studies is generally expected in meta-analyses because of the potential differences in baseline characteristics of populations, study locations, methods used by research teams, etc. [17, 18]. Although different studies’ underlying effects are conveniently modeled to have a normal distribution as a convention in the literature, this assumption should not be taken for granted [19, 20]. The presence of between-study normality depends on the choice of effect measures because effect measures are assumed exchangeable across studies [6, 21, 22], and the presence of outlying studies could make this exchangeability assumption questionable [23, 24].

Violations of the between-study normality assumption could lead to problematic meta-analytical conclusions [5, 25]. Although the non-normality might not have substantial impacts on the point estimates, it could greatly affect the interval estimates [26]. For example, if the true between-study distributions are skewed, 95% confidence intervals (CIs) of the overall effect estimates produced by commonly used meta-analysis methods could have coverage away from the nominal level of 95% [19]. Such inaccuracy in coverage could greatly affect the conclusions about the significance of treatment effects. Moreover, a group of studies may share similar treatment effects but have substantially different effects from another group of studies. The between-study distribution could be bi-modal rather than normal. It may be sensible to perform separate meta-analyses for different groups of studies instead of pooling all studies together [27]. Such non-normality challenges the generalizability of meta-analytic conclusions. In addition, non-normality caused by a few outlying studies could seriously bias meta-analytic results [23]. It is possible to remove evident outlying studies or subgroup certain studies with similar features if they are substantially different from other studies. However, the practice of removal or subgrouping might not be well-justified when it is not pre-specified in the protocol, as this could lead to “cherry-picking” favorable studies in a systematic review [28, 29].

Several methods have been proposed to test the between-study normality assumption [16, 30]. The fundamental idea is to construct study-specific standardized effect estimates, which are calculated as the differences between individual studies’ effect sizes and the overall effect size, divided by the marginal standard deviations. These standardized effect estimates are expected to be independently and identically distributed as standard normal variables. Consequently, approaches for assessing normality, such as the quantile–quantile (Q–Q) plot and statistical tests for normality, can describe the deviation from the between-study normality assumption visually and quantitively.

Considering the lack of proper assessment of the between-study normality assumption, this article empirically assesses this assumption using the Cochrane Library, a large database of systematic reviews. Our aims are three-fold. First, we will use hypothesis testing to examine the proportions of Cochrane meta-analyses with a questionable between-study normality assumption. Second, for binary outcomes, we aim to compare the validity of the between-study normality assumption among three commonly used effect measures, i.e., odds ratios (ORs), relative risks (RRs), and risk differences (RDs). Third, we will construct Q–Q plots for assessing the between-study normality and evaluate the agreement between the visual assessment by independent researchers.

## Methods

### Datasets

This study used the Cochrane Library, a large database of systematic reviews and meta-analyses, which has been used in our previous work on assessing heterogeneity and small-study effects [18, 22, 31]. Specifically, the Cochrane Library publishes and records systematic reviews on a wide range of healthcare-related topics; it generally has better data quality than non-Cochrane reviews [32, 33]. We extracted the statistical data from all systematic reviews published from 2003 Issue 1 to 2020 Issue 1. Data withdrawn from the Cochrane reviews (which may be flawed or outdated) were also excluded from our analyses. The detailed data collection procedures have been documented in our previous publications [31, 34].

Additional exclusion criteria were applied to the meta-analyses. First, like the assessment of small-study effects based on the funnel plot [35], the statistical powers of tests may be too low for distinguishing true non-normality from chance in a meta-analysis containing few studies. Therefore, we excluded meta-analyses with less than 10 studies. Second, we employed the restricted maximum-likelihood (REML) method for the random-effects model in each meta-analysis [36]. However, when the algorithm using the REML method for estimating the overall effect size could not converge in some cases, we excluded those meta-analyses from our analysis. Third, the between-study normality cannot be assessed for homogeneous meta-analyses (${\widehat{\tau }}^{2}$=0), so these meta-analyses were also excluded.

We classified the eligible meta-analyses to include both those with binary outcomes and those with non-binary outcomes (such as continuous data, survival data, and outcomes reported as generic effect sizes). For both outcomes, we obtained the originally reported study-specific effect size and its standard error in each meta-analysis. The originally reported effect measures included the (log) OR, Peto OR, (log) RR, or RD for binary outcomes and the mean difference, standardized mean difference, and rate ratio (of count or survival data) for non-binary outcomes. For binary outcomes, we additionally extracted the counts of events and non-events in the treatment and control groups (i.e., 2 × 2 table) for each study.

### Assessing the between-study normality assumption

We used the methods recently proposed by Wang and Lee [30] to assess the between-study normality assumption in the meta-analyses. Specifically, this assumption was assessed both visually and quantitatively. The visual assessment was based on the Q–Q plot of standardized effect estimates, and the quantitative assessment was based on the Shapiro–Wilk (SW) test for normality [37]. Considering the relatively low statistical power of tests for normality, we set the significance level to 0.1. This follows the conventions for handling underpowered tests that also occur in the assessments of heterogeneity and publication bias [38, 39], although we acknowledge that the choice of the significance level is debated broadly in scientific communities [40–42].

We applied the SW test to the *originally reported* effect sizes in each meta-analysis. If the resulting *P*-value was < 0.1, then the null hypothesis of normality between studies was rejected. We recorded the test results’ statistical significance. Additionally, for each meta-analysis with a binary outcome, we used the 2 × 2 tables to re-calculate individual studies’ ORs, RRs, and RDs and applied the SW test to compare the normality assessments among these *re-calculated effect sizes*. Of note, the ORs and RRs were analyzed on the logarithmic scale, as in the convention of meta-analyses.

### Approximate proportion of truly non-normal meta-analyses

The above procedure gave the proportion of meta-analyses with significant non-normality by the SW test, denoted by $q$. Due to type I and II errors, a *P*-value < 0.1 or ≥ 0.1 did not ascertain that the between-study normality does not hold or holds in a meta-analysis. Thus, $q$ did not represent the proportion of *truly* non-normal meta-analyses, denoted by $p$.

Based on the available information, we proposed a method to approximate $p$ from $q$ as follows. By conditional probabilities, the proportion of meta-analyses with significant non-normality should be $q=p\cdot \mathrm{power}+\left(1-p\right)\cdot \alpha$, where $\alpha$ is the type I error rate of 0.1, and the SW test’s power could be determined by the simulations in Wang and Lee [30]. The statistical power depends on many factors, including the number of studies in a meta-analysis and the true between-study distributions. There is no explicit formula to calculate this power; we used the empirical evidence from simulation studies by Wang and Lee [30] to impute the SW test’s power. Based on the foregoing observations, we approximated the proportion of truly non-normal meta-analyses as $p=\left(q-\alpha \right)/\left(\mathrm{power}-\alpha \right)$. Here, we assumed that all meta-analyses were independent and shared the same power of the SW test. Although these assumptions are unrealistic, they could provide a rough proportion of truly non-normal meta-analyses for a possible range of power.

### Subgroup analyses

Methods for assessing the between-study normality assume the within-study normality. This within-study normality assumption generally requires large sample sizes and event rates that are away from the boundary values of 0% and 100% [5]. Therefore, we conducted subgroup analyses by categorizing the meta-analyses by sample sizes (for both types of outcomes) and event rates (for binary outcomes only). In each subgroup, the meta-analyses were restricted to those with studies that meet a sample size threshold, which was set to 0, 10, …, and 100. Meta-analyses with binary outcomes were further categorized based on the crude event rate, which was calculated by dividing the total event count by the total sample size across studies. The thresholds of crude event rates were set to 0–100%, 1–99%, …, and 25–75%. Of note, we did not use two-dimensional analyses with a factorial design that would lead to too many subgroups. Instead, the subgroups were created by matching the 11 thresholds of crude event rates with the foregoing 11 thresholds of sample sizes accordingly; the within-study normality assumption was gradually more likely to hold in these subgroups.

### Visual assessment of Q–Q plots

As the SW test has low statistical power for meta-analyses with a small or moderate number of studies, visual assessments of the normality based on Q–Q plots remain essential. Two authors (ZL and FMAA) independently performed visual assessments of the between-study normality in Q–Q plots of the originally reported effect sizes. To reduce workload, we focused on the meta-analyses with non-significant test results (*P*-values ≥ 0.1), i.e., when the SW test failed to detect non-normality. The two authors also assessed the Q–Q plots based on the (log) OR, (log) RR, and RD for each meta-analysis with a binary outcome.

To describe our visual assessment of normality, we set five tail scores ($-$2, $-$1, 0, 1, and 2) for tails in a Q–Q plot, representing an apparently light tail, slightly light tail, approximately normal tail, slightly heavy tail, and apparently heavy tail, respectively. Here, light and heavy tails were defined based on the normal distribution’s tails. A Q–Q plot with both light left and right tails implied a light-tailed distribution, that with both heavy left and right tails implied a heavy-tailed distribution, that with a heavy left tail and a light right tail implied a left-skewed distribution, and that with a light left tail and a heavy right tail implied a right-skewed distribution.

The normality assumption could also be affected by subgroup effects, where different subgroups may come from different distributions, leading to an overall multimodal distribution if the subgroups are inappropriately combined in the same meta-analysis. We set three mode scores (0, 1, and 2) for assessing the multimodal status, representing apparent multimodal, suspicious multimodal, and approximately unimodal distributions, respectively. Additional file 1: Figs. S1 and S2 give examples of Q–Q plots in different scenarios.

A meta-analysis was considered approximately satisfying the between-study normality assumption only if both tail and mode scores of visual assessments equal 0 in a Q–Q plot. Cohen’s $\kappa$ statistic was used to quantify the agreement between the visual assessments by the two authors [43]. We calculated Cohen’s $\kappa$ statistics for two types of assessment for: (I) all 5 × 5 × 3 = 75 categories for the 5 scores for the left tail, 5 scores for the right tail, and 3 scores for the multimodal status, and (II) 2 aggregate categories of normality (all scores equal 0) vs. non-normality (any score does not equal 0). The first type of assessment involves detailed scores evaluated by the two assessors, while the second type of assessment represents the goal of making a binary decision of whether the between-study normality assumption holds approximately.

## Results

### Characteristics of included meta-analyses

Additional file 1: Fig. S3 presents the flow chart of selecting the meta-analyses from the Cochrane Library. We collected a total of 107,140 meta-analyses, of which 64,929 had binary outcomes and 42,211 had non-binary outcomes. Among the 64,929 meta-analyses with binary outcomes, 6162 meta-analyses contained at least 10 studies. Based on their originally reported effect measures, 259 had convergence issues with the REML method, and 1669 had zero between-study variance estimates. As a result, 4234 meta-analyses were eligible for our analyses. Among the 4234 meta-analyses, 498 originally used ORs, 3340 used RRs, 32 used RDs, and the remaining used other effect measures such as Peto ORs. We re-calculated the ORs, RRs, and RDs using the 2$\times$2 tables; Table 1 presents the number of eligible meta-analyses based on the REML method’s convergence and ${\widehat{\tau }}^{2}$>0 criterion using the re-calculated ORs, RRs, and RDs for the 6162 meta-analyses with ≥10 studies.

**Table 1:** Selections of eligible meta-analyses with at least 10 studies from the Cochrane Library

	Binary outcome (*N* = 6162)				Non-binary outcome (*N* = 4014)
	Original^a^	OR^b^	RR^b^	RD^b^	Original^a^
REML failing to converge^c^	259	273	272	217	101
${\widehat{\tau }}^{2}$=0^d^	1669	1678	1719	1219	480
Eligible meta-analyses	4234	4211	4171	4726	3433

^a^Originally reported effect measures in Cochrane meta-analyses

^b^Odds ratios, relative risks, and risk differences re-calculated from 2 $\times$ 2 tables for meta-analyses with binary outcomes

^c^The restricted maximum-likelihood algorithm failed to converge

^d^The between-study variance estimate was 0

For the 42,211 meta-analyses with non-binary outcomes, 4014 meta-analyses contained at least 10 studies, of which 101 had convergence issues with the REML method and 480 had zero between-study variance estimates. Thus, 3433 meta-analyses had ${\widehat{\tau }}^{2}$>0 based on the REML method.

### Test results for originally reported effect measures

The overall proportion of meta-analyses of binary outcomes having significant non-normality between studies was 15.7% (95% CI, 14.6% to 16.8%) based on originally reported effect measures. The overall proportion of meta-analyses with non-binary outcomes having significant non-normality between studies was 26.2% (95% CI, 24.8% to 27.7%).

**Fig. 1:**
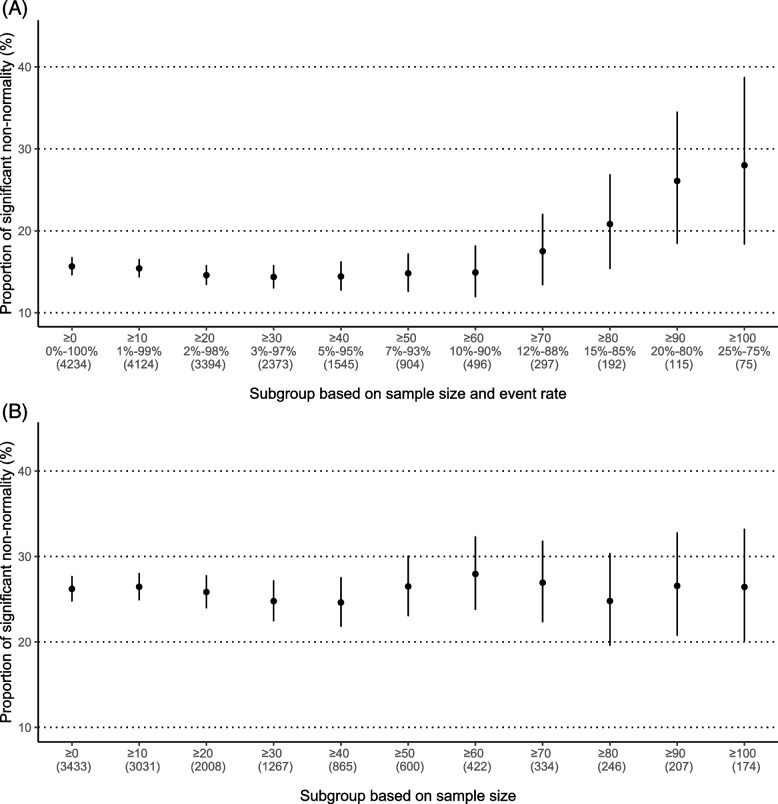
Proportions of meta-analyses with significant non-normality in different subgroups for **A** meta-analyses with binary outcomes and** B** those with non-binary outcomes. On the horizontal axis, the first line presents restrictions on study sample sizes, and the second line of panel A presents restrictions on event rates. The corresponding numbers of meta-analyses satisfying the restrictions are in parentheses. The vertical bars represent 95% confidence intervals of the proportions

We also calculated these proportions categorized by sample sizes and event rates, as shown in Fig. 1. For binary outcomes, the proportion with significant non-normality increased as the sample size increased and the event rate moved away from 0 and 100% (Fig. 1A). As the within-study normality assumption was more likely violated for smaller sample sizes and event rates close to 0% or 100%, this increasing trend implied that the potential violation of the within-study normality might confound the assessment of the between-study normality, possibly through the impact on the test power. In contrast, the proportions for non-binary outcomes were stable (Fig. 1B). This might be because most such meta-analyses used mean differences as effect measures, which converged quickly to normality within studies, even for moderate sample sizes, making the within-study normality assumption generally valid.

**Fig. 2:**
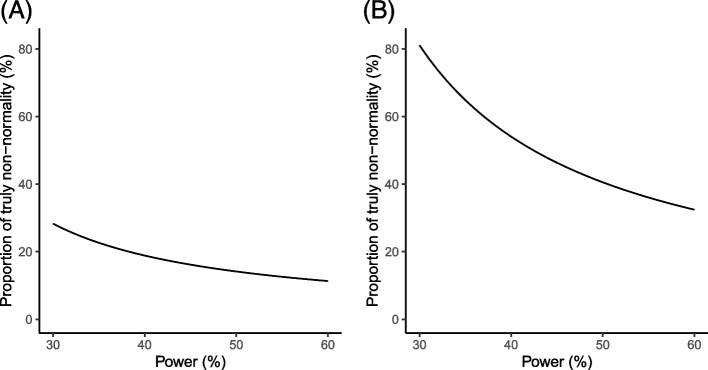
Approximate proportions of truly non-normal meta-analyses with **A** binary outcomes and **B** non-binary outcomes when the statistical power varies from 30 to 60%

According to Wang and Lee [37], the statistical power of the SW test is higher for meta-analyses with more studies. In our analyses, the median number of studies in meta-analyses was 15; the simulation studies by Wang and Lee [37] indicated that the test’s power was about 30–60%. Based on these observations and the calculation in the methods section, Fig. 2 presents the approximated proportions of truly non-normal meta-analyses. When the power of the SW test changed from 30 to 60%, the proportion for binary outcomes roughly varied from 28 to 10%, and that for non-binary outcomes roughly varied from 80 to 30%. The proportion of truly non-normal meta-analyses had a wide range, but it sufficiently suggested that the non-normality issue occurred quite frequently, especially for non-binary outcomes.

### Impact by effect measures for binary outcomes

For binary outcomes, we investigated how the choices of effect measures affected the assessment of the between-study normality. Based on the re-calculated ORs, RRs, and RDs from 2 $\times$ 2 table data among all eligible meta-analyses (Table 1), the proportions of meta-analyses with significant non-normality for ORs, RRs, and RDs were 15.1% (95% CI, 14.0% to 16.2%), 15.2% (95% CI, 14,1% to 16.3%), and 21.8% (95% CI, 20.6% to 23.0%), respectively.

**Fig. 3:**
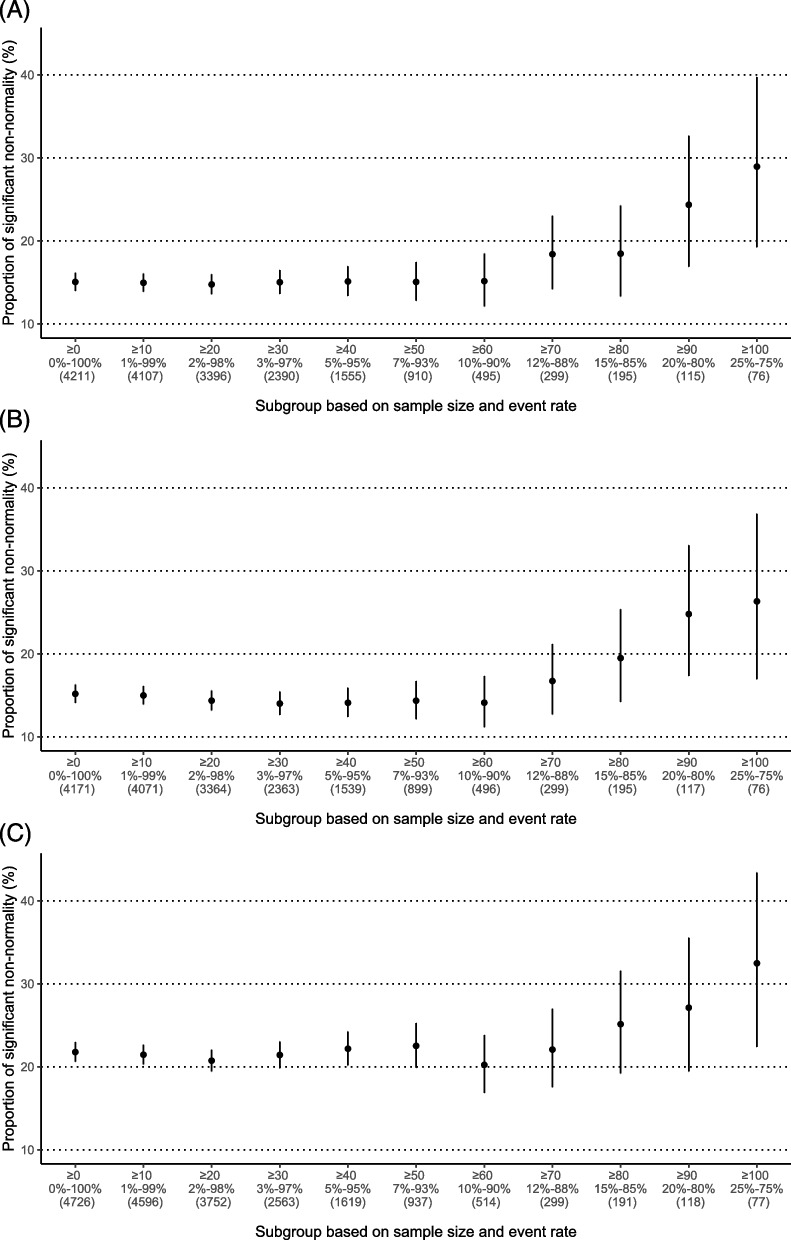
Proportions of significant non-normality in different subgroups for meta-analyses when using **A** ORs, **B** RRs, and **C** RD*s*. On the horizontal axis, the first line presents restrictions on sample sizes, and the second line presents restrictions on event rates. The corresponding numbers of meta-analyses satisfying the restrictions are in parentheses. The vertical bars represent 95% confidence intervals of the proportions

**Fig. 4:**
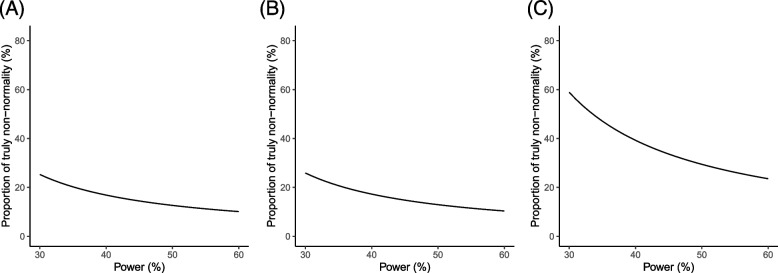
Approximate proportions of truly non-normal meta-analyses for **A** ORs, **B** RRs, and **C** RDs when the statistical power varies from 30 to 60%

For the three effect measures, Fig. 3 presents the proportions of meta-analyses with significant non-normality subgrouped by sample sizes and event rates. The proportion for ORs varied from 15.1 to 29.0%, that for RRs varied from 15.2 to 26.3%, and that for RDs varied from 21.8 to 32.5%. The proportion of meta-analyses with significant non-normality for the re-calculated RDs was lower than that based only on the 32 meta-analyses originally using the RD. This difference was likely because of sampling variability, as using all eligible meta-analyses led to much more precise results. Like the trend in Fig. 1A, the proportions were higher for larger sample sizes and event rates away from 0 and 100%. This again suggested that the within-study normality might not be valid for smaller study sample sizes or event rates closer to boundary values; this could affect the assessment of the between-study normality. Moreover, we approximated the proportions of truly non-normal meta-analyses when using ORs, RRs, and RDs (Fig. 4). The proportion for RDs varied in a wider range than the ORs and RRs.

### Visual assessment based on Q–Q plots

Table 2 presents Cohen’s $\kappa$ statistics of agreements on the visual assessment of Q–Q plots between the two independent assessors. All Q–Q plots and the two assessors’ scores can be accessed on the Open Science Framework [44]. Based on all 75 categories of tail scores and multimodal status scores, the $\kappa$ statistics were 0.36 for meta-analyses with binary outcomes and 0.37 for those with non-binary outcomes. When only focusing on 2 aggregate categories of normality vs. non-normality, the $\kappa$ statistics were 0.44 for meta-analyses with binary outcomes and 0.46 for those with non-binary outcomes. In general, these statistics implied fair to moderate agreements [45]. They did not differ much for different types of outcomes and different effect measures. The 2-category-based $\kappa$ statistics were larger than the 75-category-based ones. This difference was expected because it was more likely to achieve an agreement on whether a Q–Q plot reflects normality (i.e., scatter points approximately on a straight line) than to have a consensus on the magnitudes of non-normality.

**Table 2:** Cohen’s $\kappa$ statistics for assessing the normality based on the Q–Q plots

	Binary outcome (*N* = 2498^a^)				Non-binary outcome (*N* = 2533^b^)
	Original^c^	OR^d^	RR^d^	RD^d^	Original^a^
Cohen’s $\kappa$ statistic (I)^e^	0.36	0.35	0.35	0.36	0.37
Cohen’s $\kappa$ statistic (II)^f^	0.44	0.42	0.43	0.44	0.46

^a^Based on 2498 eligible meta-analyses with *P*-values ≥ 0.1 (from the SW test) and the between-study variance estimates > 0 for all of the OR, RR, and RD for binary outcomes

^b^Based on 2533 eligible meta-analyses with *P*-values ≥ 0.1 (from the SW test) and the between-study variance estimates > 0 for non-binary outcomes

^c^Originally reported effect measures in Cochrane meta-analyses

^d^Odds ratios, relative risks, and risk differences re-calculated from 2 $\times$ 2 tables for meta-analyses with binary outcomes

^e^Based on all 75 categories for the 5 scores for the left tail, 5 scores for the right tail, and 3 scores for the multimodal status

^f^Based on 2 aggregate categories of normality (all scores equal 0) vs. non-normality (any score does not equal 0)

## Discussion

In this study, we investigated the between-study normality assumption in random-effects meta-analyses based on a large-scale real-world dataset. Our findings suggested that the between-study normality assumption is questionable in a considerable number of Cochrane meta-analyses, although this assumption dominates the current meta-analytical practice.

We also found that the validity of the between-study normality assumption is relevant to the types of outcomes and effect measures. In general, between-study non-normality issues are less likely to occur with ORs and RRs than RDs and effect measures for non-binary outcomes. This is generally expected because RD values are bounded between $-$1 and 1, so assuming them to follow a normal distribution may not be plausible. Researchers should carefully account for the exchangeability across studies when choosing the effect measure in a meta-analysis [6, 22, 46–48].

In addition, we evaluated the confounding effects of the within-study non-normality on assessing the between-study normality by subgroup analyses with restrictions on sample sizes and event rates. For binary outcomes, the subgroup analyses showed that the between-study non-normality occurred more frequently in meta-analyses with larger sample sizes and event rates away from the boundary values of 0% and 100%. In such cases, the within-study normality was more likely valid and possibly led to a larger power of the SW test. Restricting to large sample sizes within studies generally did not affect the assessment of the between-study normality for non-binary outcomes.

Our findings suggested that this visual tool could be very subjective, as the agreement between two independent assessors was only fair to moderate. As statistical tests for normality have relatively low powers, particularly when the number of studies is small [30], the Q–Q plot remains essential for assessing normality. Nevertheless, researchers should expect high uncertainties in the conclusions of visual assessments. Such conclusions should be evaluated and discussed with multiple assessors.

**Fig. 5:**
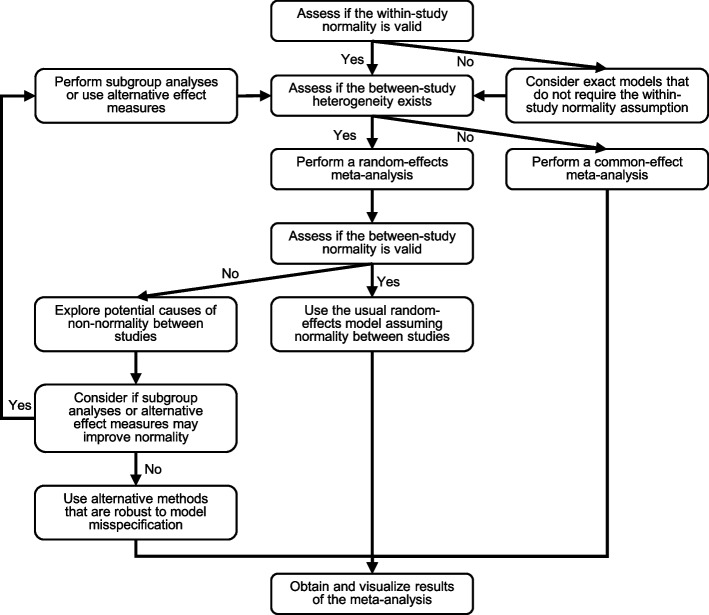
A framework of recommendations for performing a meta-analysis based on the assessments of heterogeneity and normality

Considering that the between-study non-normality is a common issue, we have some recommendations as follows. First, if there are a sufficient number of studies (e.g., > 10) and heterogeneity likely exists between studies, researchers should validate the normality assumption for performing a random-effects meta-analysis. Second, if the between-study normality in a meta-analysis may not hold, researchers should explore potential clinical characteristics of included studies that might contribute to the non-normality. For example, based on the studies’ characteristics, researchers may consider subgroup analyses, meta-regressions, and sensitivity analyses that exclude outlying studies. Small-study effects could also lead to skewed between-study distributions, so methods that account for small-study effects may be used to examine if they might improve the normality [49]. Third, researchers should consider if it makes sense to assume the effect measure is exchangeable across studies [21, 22]. If not, they may try using other effect measures to examine whether the normality could be improved. Finally, researchers may consider alternative statistical meta-analytic methods that are robust to model misspecification [50–53], non- or semi-parametric methods [54–56], and exact models that do not require the within-study normality assumption [57–59]. If the between-study normality is evidently violated, the robust methods could produce less biased results, while they may sacrifice statistical power for finding true treatment effects. Figure 5 describes a framework of recommendations based on the assessments of heterogeneity and normality.

This study had several limitations. First, due to the nature of large-scale analyses, it was not feasible to investigate the non-normality on a case-by-case basis. For example, although we might identify multimodal patterns in the Q–Q plot of a meta-analysis, we did not further investigate if such patterns were caused by certain effect modifiers or some outlying studies. When the between-study normality is violated in a particular meta-analysis, we recommend exploring the potential causes of non-normality. Second, the statistical tests for non-normality have relatively low power, and many factors could affect the assessment of the between-study normality. Those factors may include type I and II error rates of the SW test, sample sizes, and event rates. Nevertheless, many other factors (e.g., publication bias) could not be accurately taken into account. Third, although the REML method is generally recommended for estimating the between-study variance [36], it could have convergence problems that lead to a loss of about 2.5–4.4% of meta-analysis samples and thus affect their representativeness. Fourth, our analyses were restricted to meta-analyses with at least 10 studies due to the relatively low power of statistical tests for normality. This restriction is similarly recommended when using statistical methods to assess small-study effects [35]. Nevertheless, meta-analyses with a small number of studies could also seriously suffer from non-normality issues, which were not investigated in the current study. Last, the Q–Q plots were assessed by two authors, who are well-trained statisticians. The $\kappa$ statistics’ interpretations only represent the agreements between these two assessors, and they may not be generalizable to other systematic reviewers.

## Conclusions

In conclusion, despite its popularity, the between-study assumption should not be taken for granted in meta-analyses. It needs to be carefully assessed; if it is evidently violated, alternative meta-analysis methods that do not make this assumption should be considered.

## Supplementary Information

**Additional file 1: Figure S1.** Examples of Q–Q plots in four tail scenarios with respect to the normal distribution’s tails. **Figure S2.** Examples of Q–Q plots in unimodal and bimodal scenarios. **Figure S3.** Flow chart of selecting the meta-analyses from the Cochrane Library.

## Data Availability

The data are available upon reasonable request from the corresponding author.

## Abbreviations

CI: Confidence interval
OR: Odds ratio
Q–Q plot: Quantile–quantile plot
RD: Risk difference
REML: Restricted maximum-likelihood
RR: Relative risk
