# Supplementary material for: The normality assumption on between-study random effects was questionable in a considerable number of Cochrane meta-analyses
Source: BMC Med. 2023 Mar 29;21:112. doi: 10.1186/s12916-023-02823-9 (PMC10053115; doi:10.1186/s12916-023-02823-9)
Supplement: Supplementary file 1 — Additional file 1: Figure S1. Examples of Q–Q plots in four tail scenarios with respect to the normal distribution’s tails. Figure S2. Examples of Q–Q plots in unimodal and bimodal scenarios. Figure S3. Flow chart of selecting the meta-analyses from the Cochrane Library. [file 12916_2023_2823_MOESM1_ESM.pdf]

Additional file 1

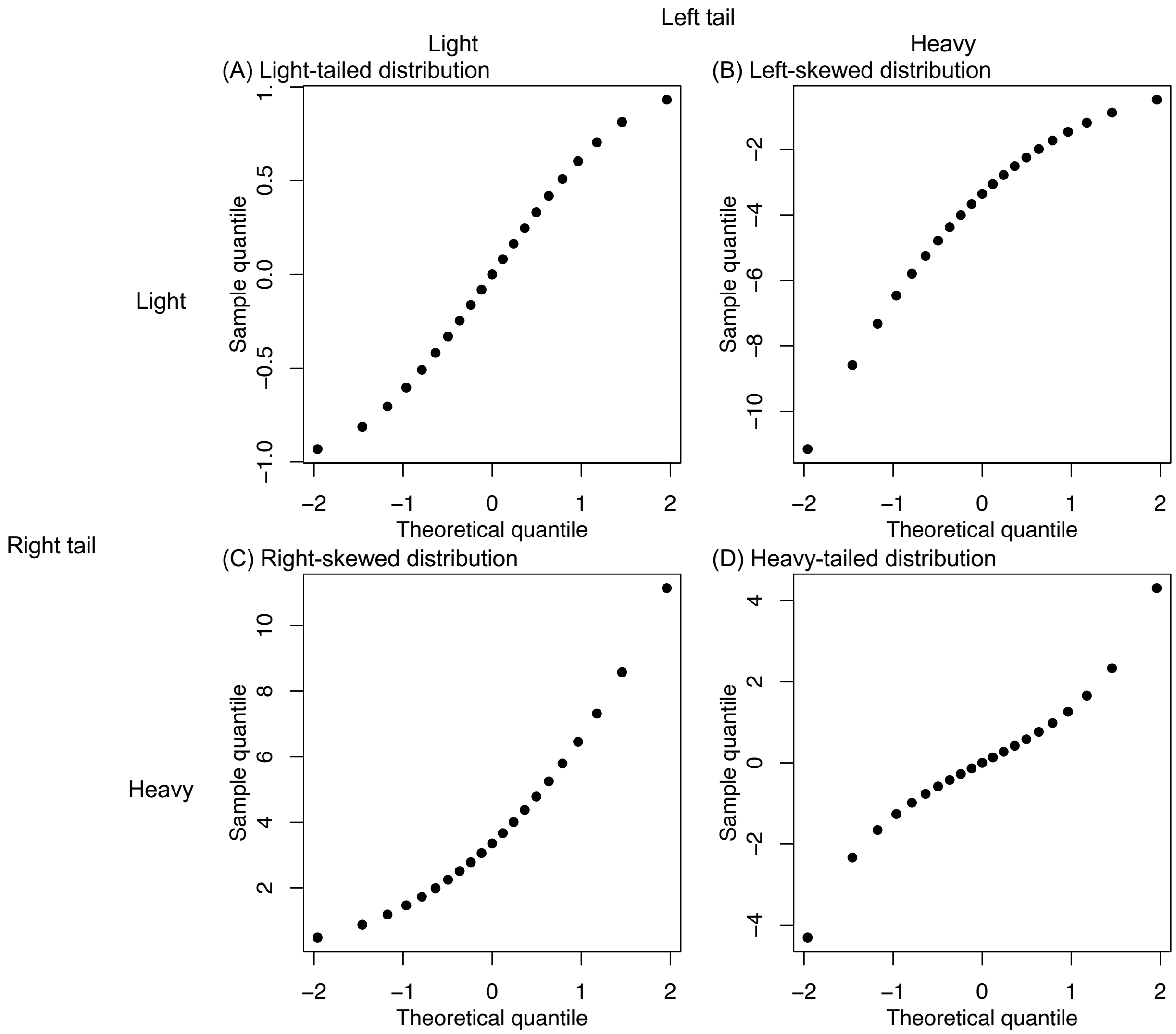

Fig. S1. Examples of Q–Q plots in four tail scenarios with respect to the normal distribution's tails. (A) Truncated normal distribution with mean 0 and variance 1, truncated within the interval (0, 1). (B) Distribution of the negative value of a gamma variable with shape and scale parameters both equal 2. (C) Gamma distribution with shape and scale parameters both equal 2. (D) t_2 distribution.

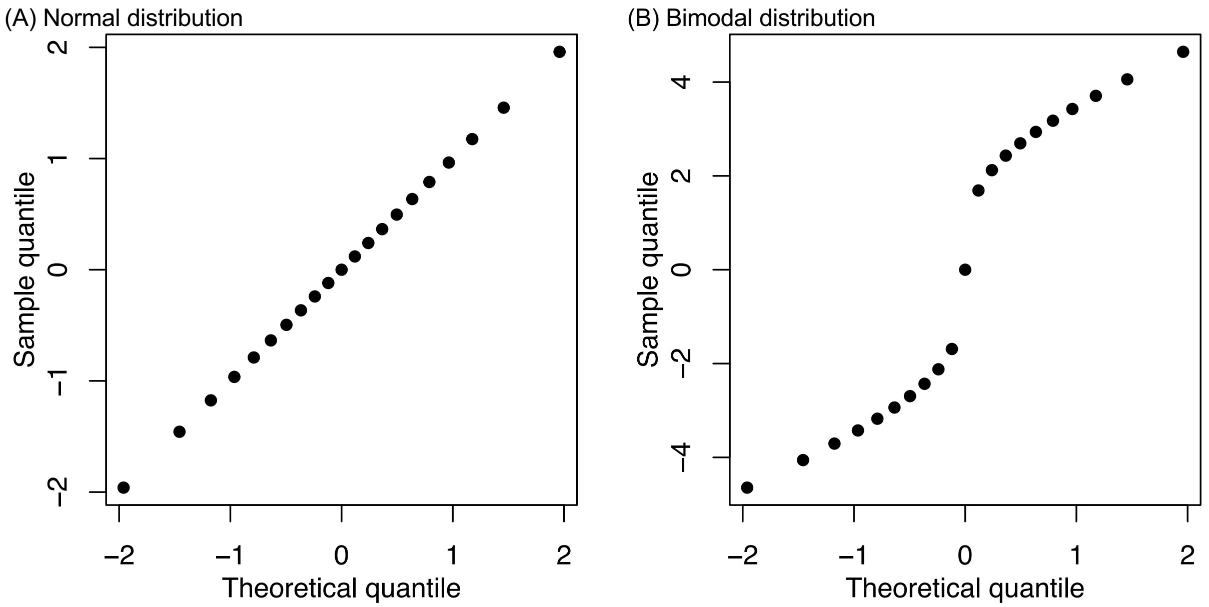

Fig. S2. Examples of Q–Q plots in unimodal and bimodal scenarios. (A) The standard normal distribution. (B) The mixture of two normal distributions, $0.5N(-3, 1) + 0.5N(3, 1)$.

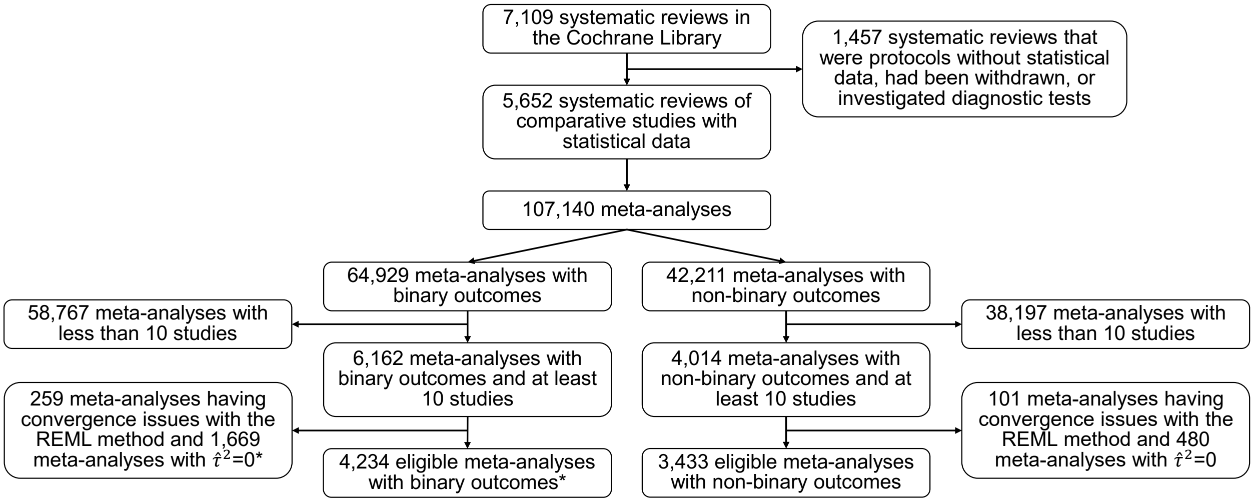

*Based on originally reported effect measures

Fig. S3. Flow chart of selecting the meta-analyses from the Cochrane Library.
